# A multi-method characterization of Elasmobranch & Cheloniidae communities of the north-eastern Red Sea and Gulf of Aqaba

**DOI:** 10.1371/journal.pone.0275511

**Published:** 2022-09-30

**Authors:** Francesco Garzon, Collin T. Williams, Jesse E. M. Cochran, Lyndsey K. Tanabe, Ameer Abdulla, Michael L. Berumen, Thamer Habis, Paul A. Marshall, Mattie Rodrigue, Lucy A. Hawkes

**Affiliations:** 1 MarAlliance, Ancon, Panama City, Panama; 2 Hatherley Laboratories, College of Life and Environmental Sciences, University of Exeter, Exeter, United Kingdom; 3 Division of Biological and Environmental Science and Engineering, Red Sea Research Center, King Abdullah University of Science and Technology, Thuwal, Kingdom of Saudi Arabia; 4 Saudi Water Sports Federation, Kingdom of Saudi Arabia; 5 OceanX, New York, NY, United States of America; Hawaii Pacific University, UNITED STATES

## Abstract

The Red Sea is particularly biodiverse, hosting high levels of endemism and numerous populations whose extinction risk is heightened by their relative isolation. Elasmobranchs and sea turtles have likely suffered recent declines in this region, although data on their distribution and biology are severely lacking, especially on the eastern side of the basin in Saudi Arabian waters. Here, we present sightings of elasmobranchs and sea turtles across the north-eastern Red Sea and Gulf of Aqaba collected through a combination of survey methods. Over 455 survey hours, we recorded 407 sightings belonging to 26 elasmobranch species and two sea turtle species, more than 75% of which are of conservation concern. We identified 4 species of rays and 9 species of sharks not previously recorded in Saudi Arabia and report a range extension for the pink whipray (*Himantura fai*) and the round ribbontail ray (*Taeniurops meyeni*) into the Gulf of Aqaba. High density of sightings of conservation significance, including green and hawksbill sea turtles and halavi guitarfish were recorded in bay systems along the eastern Gulf of Aqaba and the Saudi Arabian coastline bordering the north-eastern Red Sea, and many carcharhinid species were encountered at offshore seamounts in the region. Our findings provide new insights into the distribution patterns of megafaunal assemblages over smaller spatial scales in the region, and facilitate future research and conservation efforts, amidst ongoing, large-scale coastal developments in the north-eastern Red Sea and Gulf of Aqaba.

## Introduction

The Red Sea is a global centre of marine biodiversity [1,2]. As a semi-enclosed sea, its connections to adjacent ocean basins (i.e., the Indian Ocean and Mediterranean Sea) are restricted by the narrow Bab el-Mandeb Strait in the south, and the man-made Suez Canal in the north. Consequently, the Red Sea exhibits unique physical parameters (e.g. high temperature and high salinity; [3]), a high level of endemism [4–6], and distinct populations of marine taxa [7]. Despite these ecologically significant features, the Red Sea remains considerably understudied relative to other prominent regions of tropical marine biodiversity [8]. This dearth of knowledge is especially evident among megafauna and species of elevated conservation concern [8–10] and is perhaps most striking within the eastern Red Sea, where research has only recently begun to survey habitats in earnest.

Human activities are driving the decline of numerous marine megafauna populations worldwide [11,12], and the impoverishment of many of the marine ecosystems (e.g., mangroves, reefs and seagrasses) that support them [13–15]. Today, virtually no area of the global oceans remains unaffected by human influence [12,16,17]. Population declines have impacted all major taxa, including important ecosystem regulators like elasmobranchs [18–21] and sea turtles [22,23]. Large sharks have been severely depleted since large-scale commercial fishing operations began in the 1950s, so that only ~30% of their original biomass remains [18]. At the same time, sea turtles faced similar rapid declines worldwide up until the 1950s, when globally coordinated conservation efforts began [24]. Thanks to worldwide conservation programmes and legislation limiting exploitation, several populations of cetaceans, pinnipeds and sea turtles now seem to be recovering [25–27]. Duplicating these recoveries for many other species that remain under considerable threat, especially elasmobranchs [18,19], will likely require similar international cooperation and data-driven management [28]. Unfortunately, efforts to conserve megafauna and other marine species have been hindered by a lack of basic information in many areas of the global ocean [29], including the Red Sea basin.

Spatial patterns of marine megafauna diversity remain largely unexplored along much of Saudi Arabia’s coastline. Meanwhile, “gigaprojects” (large-scale areas dedicated to urban development and planned to become new centres for tourism and urban living in the country), like NEOM (www.neom.com), are being developed throughout the nation’s territory. These projects require strong scientific baselines of biotic diversity, distribution, and abundance upon which planning of human development and marine conservation can be based. Limited scientific understanding of the region’s biogeography has so far hindered efforts to evaluate the importance of local habitats to megafauna ecology. Establishing spatially explicit baselines for populations within the Red Sea is therefore an important first step toward the regional management of these species. The present study employed a multi-method approach to rapidly assess the occurrence of two particular megafaunal assemblages (elasmobranchs and sea turtles) in the northern Red Sea and Gulf of Aqaba to address the lack of information on the occurrence and distribution of these organisms. Bringing together multiple survey techniques with differing spatial distribution, effort, target habitat, and sensitivity to species can provide a more complete picture of the faunal assemblage of a region, although inherent biases and discrepancies between methods need to be accounted for. Surveys occurred largely within the boundaries of NEOM, which is a region that encompasses both land and sea, and is currently undergoing development (construction of urban infrastructure) at an unprecedented scale, including 450 km of coastline. Accordingly, the findings of this study not only serve as a baseline for future biological investigations, but contribute directly to the planning of major development activities and the conservation of marine environments.

## Methods

All research activities were conducted under permit from NEOM and ethical approval from NEOM and the College of Life and Environmental Sciences Biosciences Ethics Committee at the University of Exeter (application ID eCLESBio000327 v3.0).

### Study area

**Fig 1:**
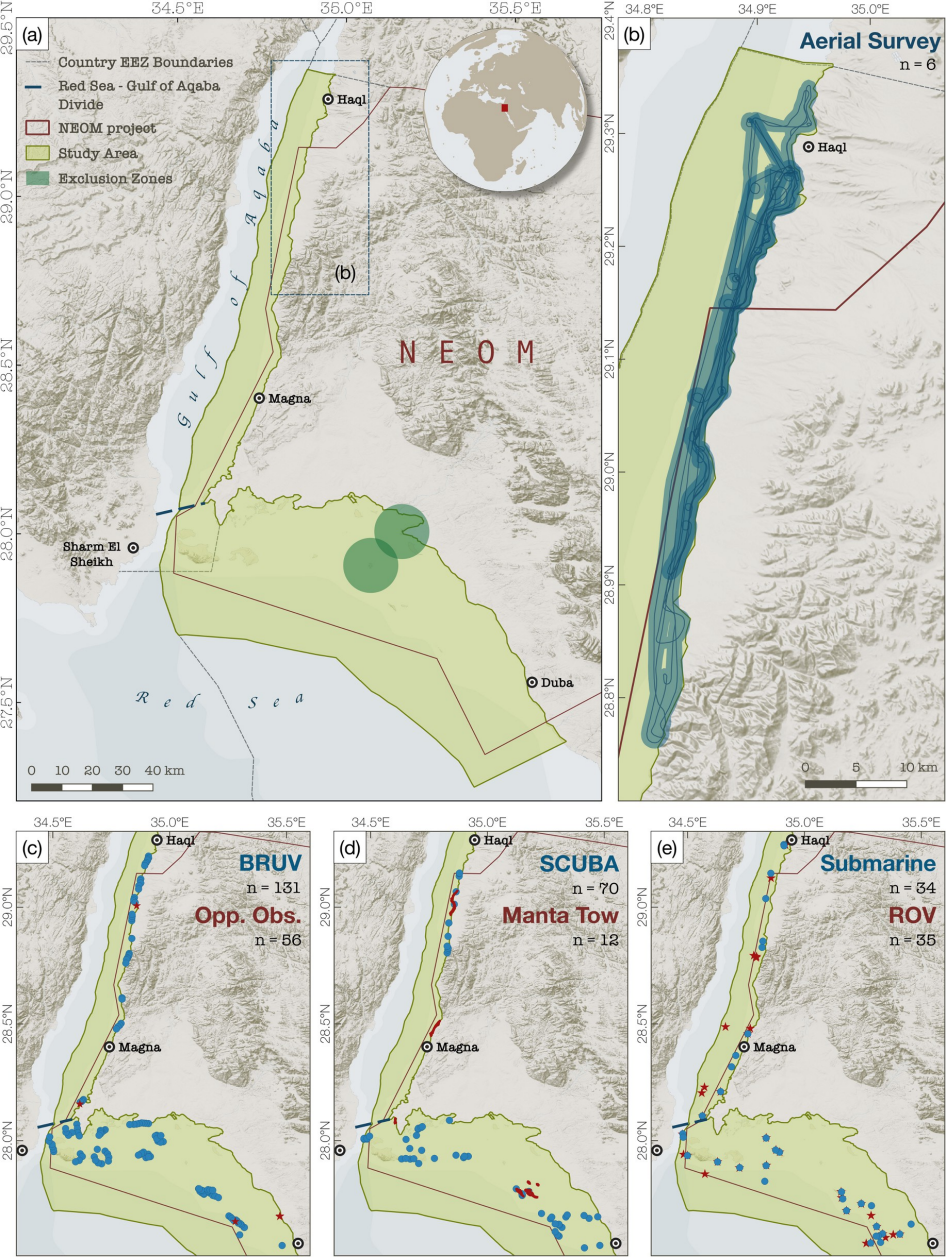
Study area and distribution of survey effort. Maps showing the study area (a, light green polygon) and the locations of survey effort, including: (b) aerial surveys (flight track in blue line and estimated survey swath in shaded blue polygon), (c) BRUV deployments and opportunistic sightings from vessels and snorkel, (d) SCUBA and manta tow surveys, and (e) ROV and submersible surveys. The number of surveys of each type are indicated as “n” in the top-right of each panel (for opportunistic sightings, n indicates the number of sightings). Green circles in (a) indicate restricted areas that were not accessible during surveys. The area covered by (b) is highlighted in (a) within the dashed blue rectangle. Base layer created in MapBox Studio (https://studio.mapbox.com) using freely available data from MapBox (hillshade and terrain data; can be found within the software) and Natural Earth (www.naturalearthdata.com; bathymetry and geopolitical contours).

During the course of six weeks (October through November 2020), the coastal and open-ocean waters bordering the Saudi Arabian side of the north-eastern Red Sea and Gulf of Aqaba (Fig 1) were extensively surveyed using a combination of techniques. The range of survey methods employed in the expedition allowed for a comprehensive assessment of all the major marine habitats of the region including coastal shallow lagoons, offshore reefs and islands, as well as epi-, meso-, and bathypelagic environments.

### Opportunistic observations

The date and location of all Elasmobranch and sea turtle species were recorded opportunistically whenever they were encountered (from the research vessel and tenders), and when conditions permitted, animals were identified to species level. Sightings were recorded by five trained experts in the various taxa and confirmed by a secondary observer whenever possible. Surveys for turtle nesting tracks were also conducted predominantly on offshore islands and, when possible, identified to species based on the track size and flipper pattern. Nesting track surveys took place on 7 separate days at Sila island, Walih Island, Yuba Island, and Al-Muwayleh (a 6 km stretch of the coast north of Duba port). The other islands in the region could not be surveyed due to coastguard permitting and logistical constraints.

### Underwater observations

Underwater observations were opportunistically collected while SCUBA diving at selected reefs and atolls within the study area (Fig 1C). SCUBA dives involved two divers swimming along linear paths following reef crests or across seagrass beds and recording sightings in front and either side of the pair to a distance of about 10m, and lasted on average 45mins. In addition, 12 ‘manta tows’ took place, where a pair of snorkellers were towed at consistent slow speed (c.a. 5 knots) on a waterski line behind a tender vessel, scanning for elasmobranchs and sea turtles to the left and right of the vessel, up to about 10m on each side of the observer.

### BRUVs

Baited Remote Underwater Video systems (BRUVs) consist of a metal frame, fitted with a 1.5 metre arm terminating in a bait pouch, placed in front of a GoPro camera (https://globalfinprint.org). BRUVs have successfully been employed to survey the abundance and diversity of multiple marine taxa in different environments [30], including elasmobranchs [31]. Although, they are not specifically employed to survey sea turtles, BRUVs also have the ability to document the occurrence of turtles when encountered[32]. During the expedition, BRUVs were deployed at 130 sites in shallow lagoon and along fringing coral reefs and islands (Fig 1C), spaced at least 500 m apart to reduce the possibility of double-counting the same animals between cameras [32]. Each BRUV was left on the seafloor at depths between 10 and 35 meters and left to record for at least 65 minutes before being retrieved. Two trained observers reviewed the entirety of the footage for each deployment, identifying organisms to the lowest taxon possible, recording time of appearance and number of individuals on the screen at any one time for each species encountered.

### Aerial surveys

Aerial surveys were conducted using a single-engine light utility helicopter (Airbus Helicopter H125). Surveys flew for 1 hour at 55 knots and 200 m altitude above the sea surface, giving an estimate swath of 700 m. Two spotters, seated rear left and right, scanned the sea surface continuously for the entire survey duration. Sea state was evaluated according to Beaufort scale at the beginning and end of the survey; all surveys took place in Beaufort state 3 or less. Sightings were called out to a dedicated data scribe, who recorded the location of observations using a handheld GPS, the presumed species, the number of individuals at each sighting, and any notes on behaviour. A fourth surveyor, seated in the front of the craft, focused solely on photographing observed animals. Species identifications were later verified post-flight.

### Deep sea observations

Deep sea observations were conducted using an Argus Mariner XL ROV, and by Triton 3300/2 MKII submersibles. ROVs and/or submersible dives lasted 3.9 hours on average and were mostly conducted near subsurface reef pinnacles or other notable geomorphological features (Fig 1E).

### Data processing

Sightings from all sources were collated into a single dataset, and the extinction threat category associated with each entry was obtained from the IUCN Red List of Endangered Species (www.iucnredlist.org). In this study, species were identified as being of “conservation concern” if they were classified by the IUCN Red List as either globally “Vulnerable”, “Endangered”, or “Critically Endangered” (IUCN assessments accessed in January 2022). The distribution of sightings was plotted in QGIS, while the total number of sightings obtained for each major taxa (Elasmobranchii and Cheloniidae) and each threat category were calculated in R [33] using custom script and plotted through the package ggplot2 [34] from the tidyverse project [35]. Because survey effort was not consistent throughout the study area, no attempt was made to derive indices of density for the species listed in this study, although we note that they would be valuable to allow for comparison with other parts of the world.

## Results & discussion

In total, 457 survey hours were carried out across the study area (Table 1 and Fig 1), including 130 BRUV deployments, 69 scuba dives, 34 submersible dives, 35 ROV dives, and 683 km of aerial surveys. Surveys covered an array of habitats from the surface to a maximum depth of 1,773 m, and included reef structures, seagrass beds, deep-water plains, and pelagic environments. A portion of the study area, stretching from Sharma Bay to Barakan Island (Fig 1A), was temporarily inaccessible during the course of the study and hence no data could be collected in this region. Dedicated surveys and opportunistic recordings yielded 407 sightings of animals of interest belonging to 28 different species, including 26 elasmobranchs and two turtle species. Overall, at least in winter months, whitetip reef sharks (*Triaenodon obesus*) and blue spotted stingrays (*Taeniura lymma*) are likely to be the most abundant elasmobranch species in the region (Table 2), while green turtles (*Chelonia mydas*) were the most often observed marine reptile (Table 2).

**Table 1:** Summary of effort for megafauna surveys in the north-eastern Red Sea and Gulf of Aqaba.

	Red Sea			Gulf of Aqaba		
Survey Method (survey depth)	N Surveys	Cumulative Effort	N Sightings	N Surveys	Cumulative Effort	N Sightings
BRUV (10 to 35 m)	89	97.5 hours	52	41 (29)	43.6 hours (37.9 hours)	10 (6)
SCUBA (5 to 23 m)	52	39 hours	78	17 (17)	13.5 hours (13.5 hours)	2 (2)
Submarine and ROV (0 to 1772 m)	45	163.8 hours	31	24 (10)	99.3 hours (56.4 hours)	23 (6)
Manta Tow (5 to 15 m)	6	35.3 km	29	6 (2)	71.8 km (33.2 km)	41 (31)
Aerial Survey (sea surface)	0	-	-	6 (6)	683 km (683 km)	60 (60)

The number of surveys, cumulative effort (calculated as the cumulative survey time or distance covered by the surveys), and cumulative number of sightings are listed for each survey method and partitioned between the Red Sea and Gulf of Aqaba. Numbers in brackets relate to surveys conducted in the northern Gulf of Aqaba (i.e. the area covered by aerial surveys).

**Table 2:** Summary of elasmobranch and Cheloniidae sightings in the north-eastern Red Sea and Gulf of Aqaba.

Order/Superorder	Family	Species	Status	NRS	GOA	Total count
*Chondrichthyes*						
Batoidea	Aetobatidae	*Aetobatus ocellatus*	VU	13	4	17
	Dasyatidae	*Himantura fai* ^1^	VU	1	3	4
		*Himantura uarnak*	EN	2	2	4
		*Pastinachus sephen*	NT	5	4	9
		*Taeniura lymma*	LC	25	13	38
		*Taeniurops meyeni* *^1^	VU	0	1	1
		*Urogymnus granulatus*	VU	2	0	2
	Myliobatidae	*Mobula birostris* *	EN	1	0	1
		*Mobula thurstoni* *	EN	1	0	1
	Rhinobatidae	*Glaucostegus halavi*	CR	5	0	5
		*Rhinobatos punctifer* *	NT	2	0	2
Selachimorpha	Alopiidae	*Alopias pelagicus* *	EN	2	1	3
	Carcharhinidae	*Carcharhinus albimarginatus*	VU	14	0	14
		*Carcharhinus amblyrhynchos* ^1^	EN	3	1	4
		*Carcharhinus brevipinna* *^1^	VU	1	1	2
		*Carcharhinus longimanus* *	CR	3	0	3
		*Carcharhinus melanopterus* *	VU	9	0	9
		*Carcharhinus plumbeus* *	EN	0	3	3
		*Carcharhinus sorrah* *	NT	1	0	1
		*Galeocerdo cuvier* *	NT	1	1	2
		*Triaenodon obesus*	VU	32	1	33
	Ginglymostomatidae	*Nebrius ferrugineus*	VU	3	0	3
	Rhincodontidae	*Rhincodon typus*	EN	4	1	5
	Sphyrnidae	*Sphyrna lewini*	CR	4	0	4
	Stegostomatidae	*Stegostoma fasciatum*	EN	1	0	1
	Triakidae	*Iago omanensis* *	LC	6	19	25
*Reptilia*						
Testudines	Cheloniidae	*Chelonia mydas*	EN	135	79	214
		*Eretmochelys imbricata*	CR	15	8	23

List includes number of individuals sighted for each species (count) in the two regions (NRS = Northern Red Sea, and GOA = Gulf of Aqaba) and their conservation status, according to IUCN Red List assessments (LC = Least Concern, NT = Near Threatened, VU = Vulnerable, EN = Endangered, CR = Critically Endangered).

* New positional record for the species in Saudi Arabia.

^*1*^ New record for the species in the Gulf of Aqaba.

### Elasmobranchs

**Fig 2:**
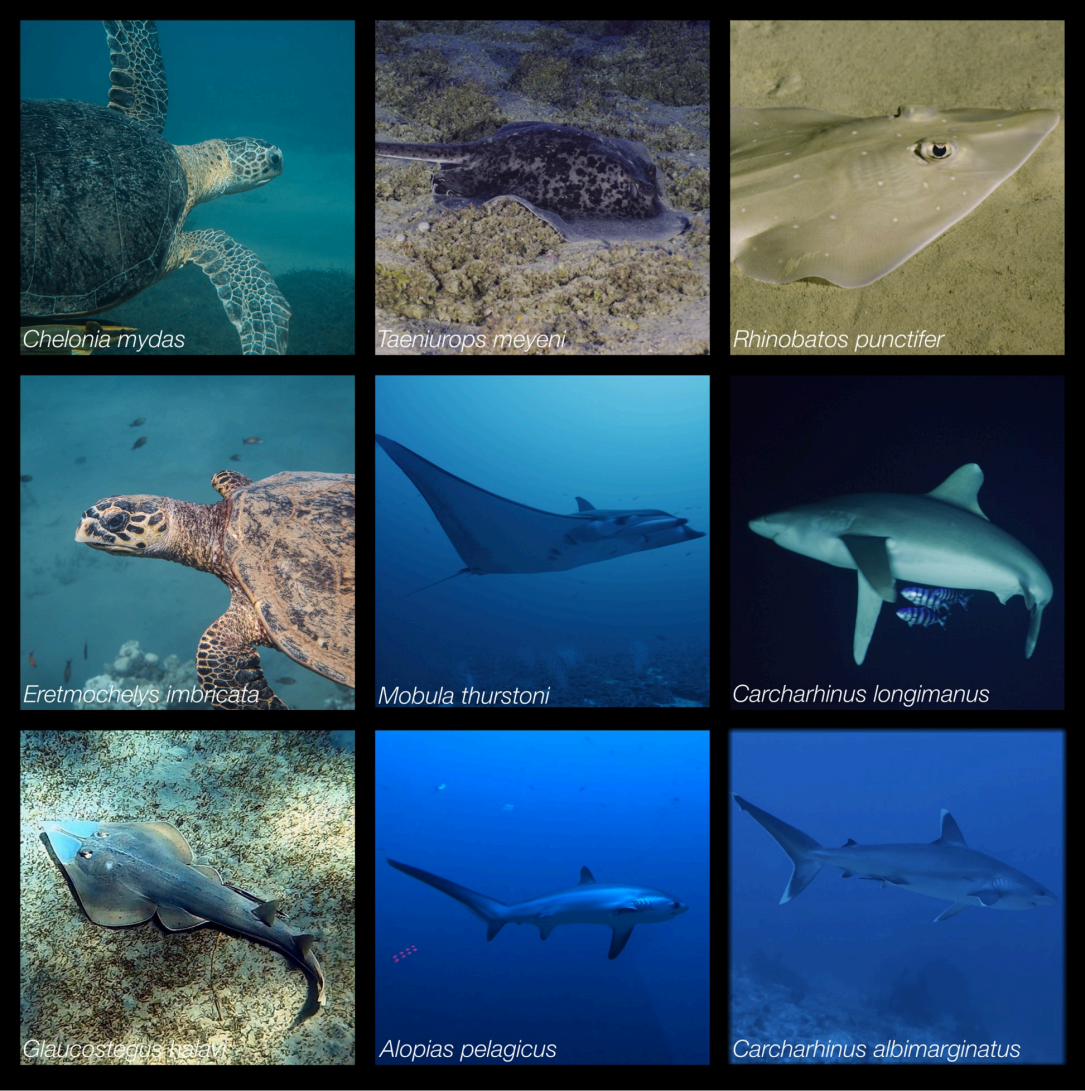
Example images of marine megafauna sightings across the expedition. Sightings including (left-to right, top to bottom): Green turtle (*C*. *mydas*), round ribbontail ray (*T*. *meyeni*), spotted guitarfish (*R*. *punctifer*), hawksbill turtle (*E*. *imbricata*), bentfin devilray (*M*. *thurstoni*), oceanic whitetip shark (*C*. *longimanus*), halavi guitarfish (*G*. *halavi*), pelagic thresher shark (*A*. *pelagicus*), and silvertip shark (*C*. *albimarginatus*).

Most shark populations in the Red Sea remain poorly studied, and, with the exception of manta rays, there is almost no regional research on batoids [9,36,37]. Checklists of the presence or absence of many other species have been compiled [38,39], but the spatio-temporal distribution of most elasmobranchs in the Red Sea is otherwise largely undescribed. Here, we show that at least 26 species of elasmobranchs occupy the northern Red Sea and Gulf of Aqaba in the winter months, including 15 species of sharks and 11 species of rays (Table 2). While market surveys revealed a wide variety of elasmobranch species being caught in the south-eastern Red Sea [40], previous in-situ surveys of elasmobranch diversity along the Red Sea have only documented the presence of 9 species of sharks and 8 species of rays along the Saudi Arabian coastline [21,38,41]. In the present study, we add a further 9 species of sharks and 4 species of rays to these data, over a much more concentrated period, to represent the diversity of the elasmobranch assemblage in Saudi Arabia during the winter months (Table 2). New records include the bentfin devilray (*Mobula thurstoni*), oceanic manta ray (*Mobula birostris*), pelagic thresher shark (*Alopias pelagicus*; Fig 2), and tiger shark (*Galeocerdo cuvier*), which had not previously been recorded at these latitudes in the Red Sea, as well as five species in the genus *Carcharhinus* which were so far undocumented in Saudi Arabia. We furthermore record the first sightings of grey reef shark (*Carcharhinus amblyrhynchos*, but see putative observation in Naylor et al., 2012 [42]), spinner shark (*Carcharhinus brevipinna*), pink whipray (*Himantura fai*), and round ribbontail ray (*Taeniurops meyeni*) in the Gulf of Aqaba [38]. These sightings suggest a broader geographical range for *H*. *fai* and *T*. *meyeni* than previously known, extending into the Gulf of Aqaba and not limited to the Red Sea basin.

Two shark species commonly observed in the central and southern Red Sea [21,41], the silky (*Carcharhinus falciformis*) and bignose shark (*Carcharhinus altimus*), were not encountered in our surveys of the northern Red Sea. As species with similar biology and ecology were sighted, it seems unlikely that survey techniques prevented us from recording these species. The absence from our records of these species, which are considered commonplace elsewhere, may suggest latitudinal differences in species composition across the Red Sea, or preferential occupancy of different latitudes at different times of the year for these species. However, dedicated, and structured surveying will be necessary to clarify the existence of these patterns.

In addition to focusing on only a few species, elasmobranch research conducted to date is not evenly distributed throughout the Red Sea [21]. Most elasmobranch studies have been conducted over a limited spatial range, for example, near research stations such as the King Abdullah University of Science and Technology (near Jeddah, in the central Red Sea), or at known aggregation sites for whale sharks [37,43–45]. In contrast, the northern coast of Saudi Arabia has been almost entirely unstudied. To our knowledge, the surveys described herein represent the first effort to catalogue elasmobranchs to such an extent in the northern Red Sea and Gulf of Aqaba. In the present study, we provide additional and, in some cases, novel positional records for 26 elasmobranchs, enabling more effective research and protection of these organisms. The data collected shows that elasmobranchs are found throughout the northern Red Sea and Gulf of Aqaba in winter, but that large-bodied sharks were mostly absent from the Gulf of Aqaba, with the exception of sporadic sightings of a single tiger shark (*Galeocerdo cuvier*), spinner shark (*Carcharhinus brevipinna*), whitetip reef shark, and three sandbar sharks (*Carcharhinus plumbeus*). An overall greater diversity of elasmobranch species was encountered in the Northern Red Sea (24 species) than in the Gulf of Aqaba (13 species), and the species found in the Gulf of Aqaba were also observed in the Red Sea, with the exception of the sandbar shark and the round ribbontail ray (*Taeniurops meyeni*).

**Fig 3:**
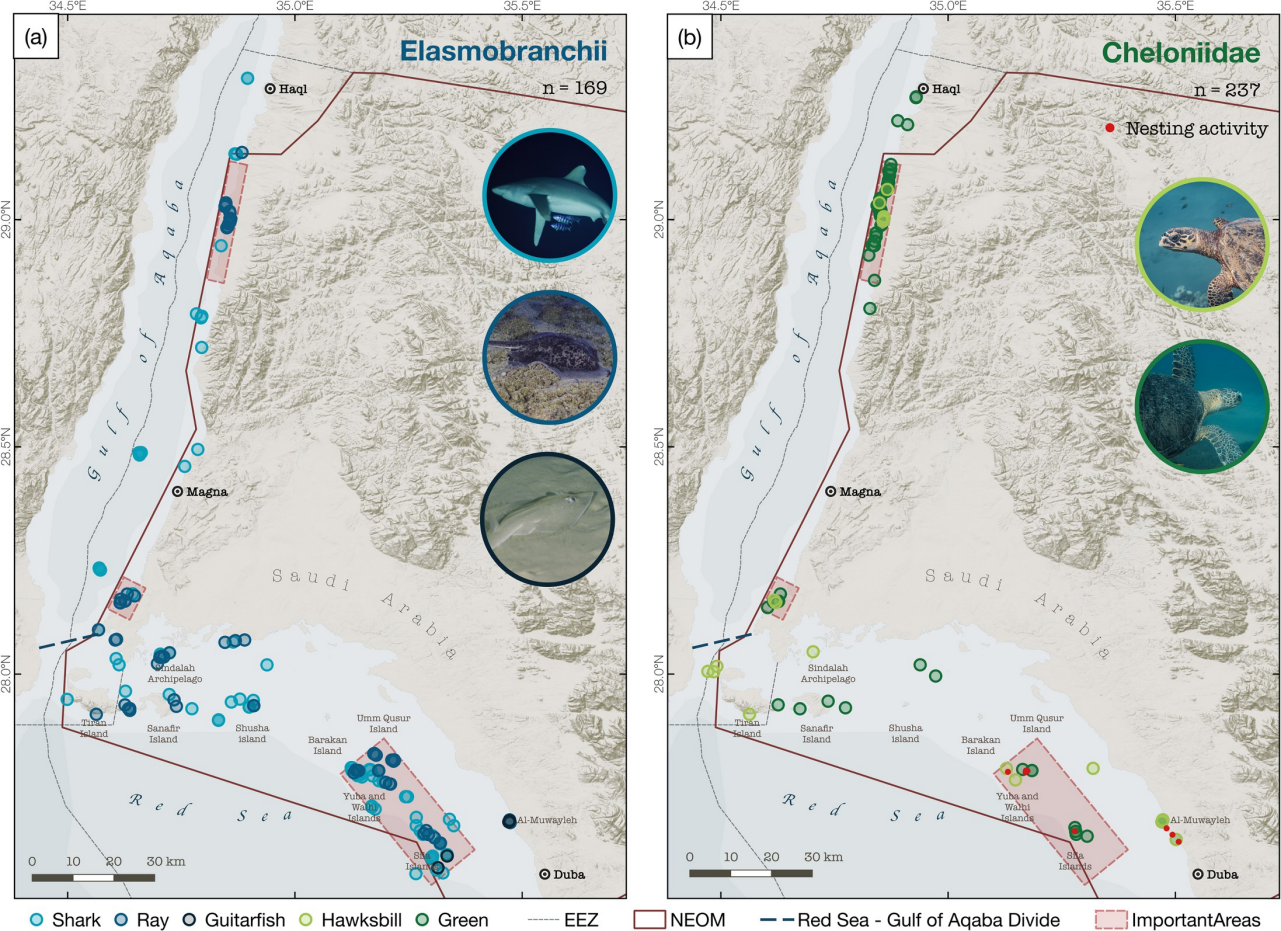
Distribution of elasmobranch and Cheloniidae sightings. Maps showing sightings of (a) elasmobranchs, (b) sea turtles. Sighting locations are differentiated by colour to indicate: Sharks (light blue), rays (dark blue), guitarfish (black), hawksbill turtle (light green), and green turtle (dark green). Locations where signs of nesting were recorded (abandoned nests or tracks on the shore) are indicated as red dots in (b) (the same symbol is applied for both hawksbill and green turtle nesting activity). The boundaries of the NEOM gigaproject are indicated by a red line, and areas of particular importance are highlighted in red rectangles. Base layer created in MapBox Studio (https://studio.mapbox.com) using freely available data from MapBox (hillshade and terrain data; can be found within the software) and Natural Earth (www.naturalearthdata.com; bathymetry and geopolitical contours).

There has been no updated assessment of the status of elasmobranchs in the Red Sea after Bonfil (2003) [39] to the authors’ knowledge, and despite a royal decree in 2008 prohibiting all shark-fishing activity in the Kingdom of Saudi Arabia, there apparently remains no appropriate fisheries enforcement. Immature sharks appear to be predominantly landed, which is likely to already be leading to recruitment overfishing [40]. The majority of the elasmobranch species sighted in NEOM waters were of global conservation concern (77%, classified by the IUCN Red List as either Vulnerable, Endangered or Critically Endangered), with three of the 26 species considered to be Critically Endangered (the halavi guitarfish, oceanic whitetip shark, and scalloped hammerhead, *Sphyrna lewini*). Scalloped hammerheads were always sighted in close proximity to reef pinnacles and were reliably encountered around a reef pinnacle in close proximity to Sila island on two separate occasions, while oceanic whitetip sharks were sighted in proximity of Tiran island and at reef pinnacles south-east of Sila island. Furthermore, particularly high numbers of the halavi guitarfish (*Glaucostegus halavi*) were encountered at Al-Muwayleh (in the south-eastern corner of the study area), within seagrass beds and lagoons located north of Duba (Fig 3A).

### Cheloniidae

In total, five species of sea turtles have been reported in the Red Sea [46], but only green (*C*. *mydas*) and hawksbill (*Eretmochelys imbricata*) sea turtles are known to nest in the region [10,47]. These were also the only two species encountered in the present surveys, and no sightings were made of the other three sea turtle species (leatherback, *Dermochelys coriacea*; olive-ridley, *Lepidochelys olivacea*; and loggerhead, *Caretta caretta*). Sea turtles were found in association with reef structures or seagrass beds near the coast and islands (Fig 3B), but never in open waters. Green turtles were most often seen in and around seagrass beds, which likely constitutes the majority of their diet in this region [48]. Al-Muwayleh in particular was found to be an important foraging ground, and has extensive seagrass meadows and numerous sightings of feeding turtles. Other sightings of green turtles were recorded around Tiran and Sanafir islands and in pockets of seagrass found along the coast of the Gulf of Aqaba (Fig 3B). Given the strong association between green sea turtles and seagrass beds [49], it is likely that the species is also found in other un-surveyed areas in the region in which seagrass is found. Sharma bay, for example, contains extensive seagrass beds, while smaller pockets can also be found in the bay between the mainland and the Sindalah archipelago and the bay systems north of it facing the Gulf of Aqaba. However, in-situ observations are necessary to confirm the presence of green sea turtles in these areas.

Hawksbill turtles were instead found in association with coral reef structures, in line with their dietary preference for sponges and soft corals [50]. Sightings of this species were also recorded both in the Red Sea and the Gulf of Aqaba, and overlapped with sightings of green turtles at Yuba and Walih islands as well as along the Gulf of Aqaba. Although turtles were recorded throughout the survey area, there was a particularly numerous aggregation of turtles in the northernmost part of NEOM waters in the Gulf of Aqaba, where up to 37 turtles were repeatedly seen close to the surface in a small survey leg of just 16.2 km by helicopter and in-water surveys. The area comprised extensive seagrass patches as well as coral reef structures, suggesting it may constitute a feeding area for the turtles. While the limited temporal scale of the survey does not inform on the residency of turtles at this site, turtles are known to display strong fidelity to their feeding grounds [51,52]. Should the presence of turtles at this site be recorded at other times of the year, the area could constitute an important site for the taxon that should be particularly considered in regional marine planning. The site would also be of great research interest, as while regionally important nesting habitats have been identified, information on the abundance and trends of turtles at foraging sites is lacking [10].

Signs of recent nesting activity of both turtle species were also observed on some of the islands surveyed in the northern Red Sea area, towards the southern border of the study area (Fig 3B). Relatively high numbers of old (already hatched and abandoned) green and hawksbill turtle nests were found at Yuba and Walhi islands (83 and 42, respectively, equivalent to 0.38 and 0.08 nests/m), belonging mostly to green turtles (99% and 95% of nests at the two sites, respectively). Assuming a mean clutch number of 5.9 for green turtles [53], and 2.74 for hawksbill turtles [54], the nests recorded suggest a minimum number of 14 green turtles and 1 hawksbill turtle nesting at Yuba island, and 3 green turtles and 1 hawksbill turtle nesting at Walih island. Previous surveys found higher nesting activity for both species at these sites, and other islands in the region are also believed to host considerable nesting activity [47]. The nesting season extends from May to October and peaking in early August for green turtles, and May to June only for hawksbill turtles [47], which is likely why few nesting tracks were observed in this study. These islands likely represent crucial sites for sea turtle conservation in the region and should be given further attention, directed especially at quantifying nesting activity and ensuring exclusion from potential future land and marine development zones.

### Notes on survey methods

Survey methods were not employed equally in the two regions due to logistical and permitting restrictions working in this challenging part of the world, which could have influenced patterns of species composition, particularly since detectability is likely not equal for all species within each method. Due to logistical constraints, aerial surveys were only conducted in the northern Gulf of Aqaba and likely preferentially recorded sightings of surface-dwelling animals such as turtles. Indeed, 24% of turtle sightings, but only 1 ray sighting, were recorded during aerial surveys, suggesting the method is more useful for turtle surveying than sharks. However, the surveys only identified 2 species (green turtle and spotted eagle ray), which had also been seen in the same region by other methods. While it seems that helicopter-based aerial surveys wouldn’t have changed the species composition observed in the two regions, they highlighted areas of interest with numerous sightings. Given the highly concentrated spatial extent of the surveys, it is also likely that other important areas exist that weren’t documented in this study. A more extensive aerial survey search of the region would serve to obtain a complete picture of the distribution and abundance of sea turtles in the region, for example. Manta tows similarly contributed 19.4% of turtle sightings and 29.8% of ray sightings, but only 1 shark sighting. In this case however, many of the species sighted during tows were not observed in other surveys and would have otherwise been missed. Overall, 59.1% of species in the Gulf of Aqaba and 40% of species in the northern Red Sea were only sighted in 1 survey method (Table 3), highlighting the importance of a multi-method approach to biodiversity monitoring.

**Table 3:** Break-down of number of species sighted by survey methods in the north-eastern Red Sea and Gulf of Aqaba.

Region (number of species)	Method	Species sighted	% Species in the region	% Total species by method	Unique species sighted	% Unique species (compared to total species in region)
NRS (30)	Aerial surveys	/	/	/	/	/
	BRUV	12	40.0	85.7	4	13.3
	Manta tows	4	13.3	57.1	1	3.3
	ROV/Submarine	9	30.0	81.8	4	13.3
	Scuba dives	10	33.3	90.9	2	6.7
	Opportunistic observations	12	40.0	100	1	3.3
GOA (22)	Aerial surveys	2	9.1	100	0	0.00
	BRUV	5	22.7	35.7	2	9.1
	Manta tows	7	31.8	100	4	18.2
	ROV/Submarine	5	22.7	45.5	4	18.2
	Scuba dives	5	22.7	45.5	2	9.1
	Opportunistic observations	4	18.2	33.3	1	4.6

For each survey region (NRS = Northern Red Sea, GOA = Gulf of Aqaba) and survey method (BRUV = Baited Remote Underwater Video, ROV = Remotely Operated underwater Vehicle), columns report the number of species sighted, the percentage of species sighted in the region observed by each method, the percentage of all species observed by a given method observed by that survey method in each region, the number of species observed solely by each methods for the region (i.e. unique species), and the percentage of all species observed in a region that the unique species represent.

In the northern Red Sea, BRUVs and opportunistic sightings yielded the highest number of species sighted (Table 3), though SCUBA and ROV/submersible surveys also identified similar numbers. In the Gulf of Aqaba, manta tows identified the highest proportion of species (31.8%), with BRUVs, SCUBA, and ROV/Submersible surveys also contributing a high proportion of species (22.7% each; Table 3). Expectedly, ROV/Submersible surveys were among the highest contributors of unique species in both regions (i.e. species not sighted in any other method), as they took place in habitats and depths not reached by other methods. Equally important were manta tows in the Gulf of Aqaba (18.1% of unique species) and BRUVs in the northern Red Sea (13.2% of unique species; Table 3). SCUBA and opportunistic surveys instead revealed few novel species not already encountered by other methods, perhaps due to these surveys covering similar habitats as BRUVs and manta tows but involving higher levels of human disturbance. Overall, the differences in species contribution among methods suggest that a multi-method approach to species sampling is necessary to obtain a more complete picture of the faunal assemblage of the region, especially when combining surveys that target different habitats (such as deep-water surveys, BRUVs, and shallow water manta tows).

### General patterns of distribution and conservation status of species in the region

Notable differences in species composition and relative frequency of sightings were observed between the Gulf of Aqaba (north of the Strait of Tiran) and the north-eastern Red Sea. More sightings of sharks and turtles were recorded overall in the northern Red Sea compared to the Gulf of Aqaba (Fig 3), with the exception of a small area south of Haql, which was found to host high numbers of green and hawksbill turtles, as well as three species of stingray (bluespotted ribbontail ray, *T*. *lymma*, cowtail stingray, *Pastinachus sephens*, and pink whipray, *Himantura fai*). Species diversity was also found to be substantially higher in the northern Red Sea (26 species) than in the Gulf of Aqaba (15 species), though a wide overlap in species composition exists between the basins. The difference in the number of sightings and diversity of animals encountered in the two basins cannot be accounted for solely by differences in survey effort (153.45 survey hours in the Gulf of Aqaba and 301.35 hours in the Red Sea). Encounter rates (sightings per unit of effort) were higher in the northern Red Sea than the Gulf of Aqaba for BRUV (0.584 vs 0.244 sightings/hour), manta tows (0.822 vs 0.571 sightings/km), and SCUBA surveys (2.000 vs 0.148 sightings/hour). The differences may be attributed to the less diverse habitat found in the Gulf of Aqaba (characterised by steeply descending seafloor) compared to the northern Red Sea, where a more varied topography has allowed for a variety of habitats (from shallow seagrass meadows to extensive coral reefs and numerous offshore, sub-surface pinnacles) [55–57].

**Fig 4:**
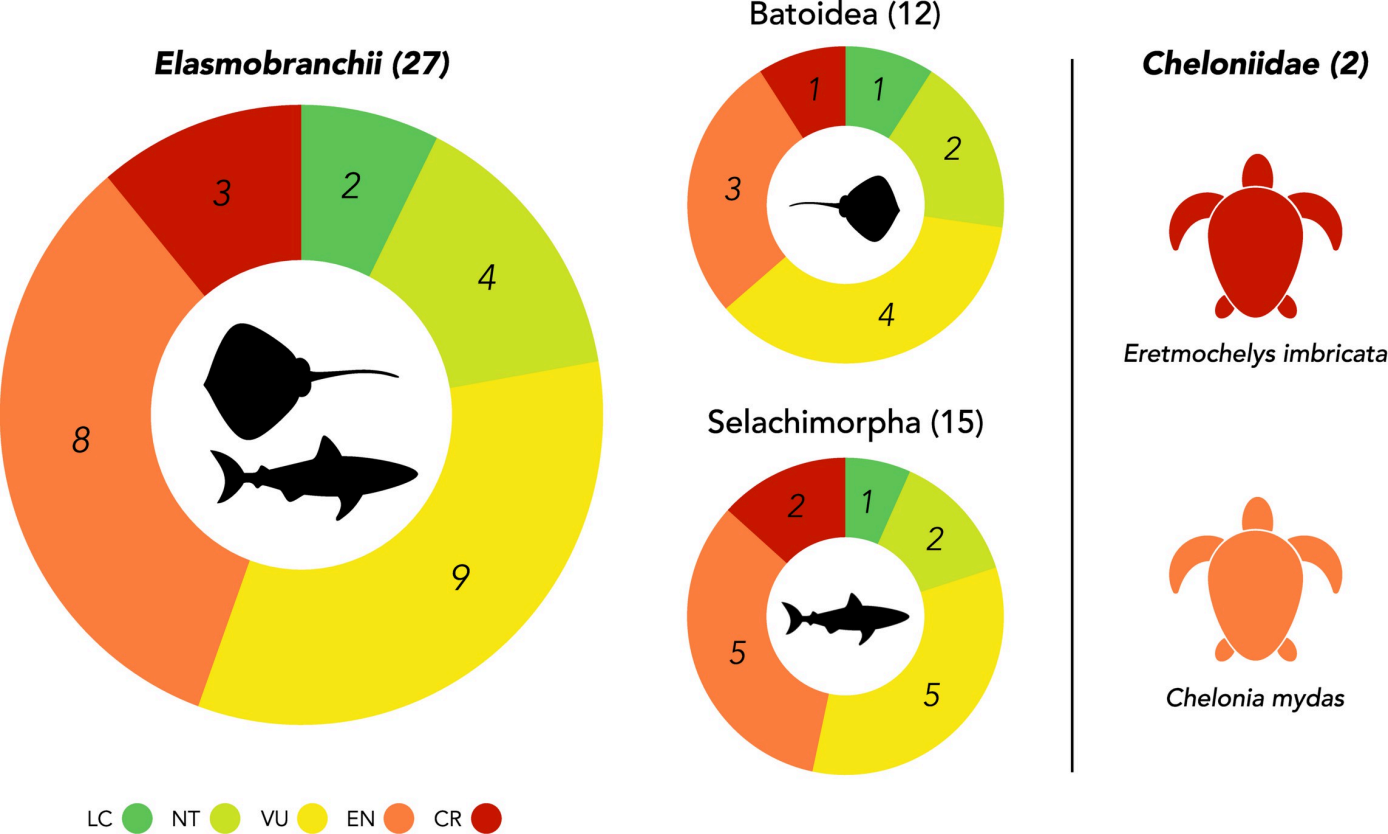
Conservation status of sighted elasmobranch and Cheloniidae species. For each main taxonomic group, conservation status is shown as percentage of encountered species that fall into each threat category (CR = critically endangered, EN = endangered, VU = vulnerable, NT = near threatened, LC = least concern, as listed on the IUCN Red List of endangered species).

The majority of the marine megafauna species sighted in NEOM’s waters were of conservation concern (22 of 28 species, 78.6%) and four were Critically Endangered (Fig 4). Both species of sea turtles sighted were of high conservation concern globally, and three of the 26 elasmobranchs sighted are considered to be Critically Endangered. The developing NEOM region plans to reserve 95% of its land and sea area for nature conservation, though restricted areas of heightened protection, and enforcement, may be necessary to foster recovery of these threatened species. Our findings suggest that particular attention should be given to two bay systems located in the north and south of the Gulf of Aqaba (Fig 3), as these areas host high numbers of sea turtle and ray species. Reef pinnacles (particularly those found near Sila island), Sila and Yuba island, and seagrass beds along the coast in the south-eastern portion of NEOM’s marine region should also be explored further for their potential to host significant number of Critically Endangered species such as the scalloped hammerhead, nesting hawksbill turtles, and halavi guitarfish, among others.

Our results highlight the potential for globally significant conservation efforts in the NEOM region. Marine megafauna are important species as both indicators of ecosystem health and as iconic targets for engaging the broader community in marine conservation [58]. The new NEOM gigaproject has committed to protecting nature and building a blueprint for sustainability as it undertakes “regenerative development” across the study area, and thus has an opportunity to design conservation management to benefit wildlife. As an independent entity, the NEOM project has the ability to directly act upon the data collected within its boundaries and enact changes in a streamlined decision-making process that can lead to effective adaptive conservation strategies. The data we provide here offer an initial baseline of the megafauna found in the region to inform future research developments, but can also be used to directly influence conservation by, for example, informing marine protected area planning, and contributing to the long-term monitoring of the health of the marine environment as development progresses.

## Supporting information

S1 File Sightings data collected in the study and used for analysis. Table of sightings contains species name, location record, methods of survey in which sighting was recorded, and conservation status of the species.
(CSV)
