# Correction: A multi-method characterization of Elasmobranch & Cheloniidae communities of the north-eastern Red Sea and Gulf of Aqaba

**DOI:** 10.1371/journal.pone.0315200

**Published:** 2024-12-04

**Authors:** The PLOS ONE Staff

The affiliation for the fifth and eighth author is incorrect. The correct affiliation is not indicated. Ameer Abdulla and Paul A. Marshall are both not affiliated with #4 but with: NEOM, Department of Nature Conservation, Tabuk, Kingdom of Saudi Arabia.

The publisher apologizes for the error.
